# Diseases of the Nose, Mouth, Pharynx and Larynx

**Published:** 1910-07

**Authors:** 


**Diseases of the Nose, Mouth, Pharynx and Larynx.** By **Alfred Bruck,** Berlin. Translated by **F. W. Forbes Ross,** M.D., Edin., F.R.C.S., Eng. Octavo, pp. 639. Illustrated. New York. Rebman Co. (Cloth, $5.00.)

This book is intended to meet the requirements of the men who are in general practice. A careful reading of its contents would indicate that nothing has been omitted by the author and translators that is of real practical value to those for whom it was intended. The work is divided into four parts—namely, diseases of the nose, of the mouth, of the throat and of the larynx. Each subject is treated in a very comprehensive manner. The anatomy and physiology of the parts are discussed at considerable length as well as the medical and surgical treatment of the entire region of the nose, mouth, throat and larynx. The size and general make-up of the book afford an attractive volume.

